# High inspired CO_2_ target accuracy in mechanical ventilation and spontaneous breathing using the Additional CO_2_ method

**DOI:** 10.3389/fmed.2024.1352012

**Published:** 2024-05-22

**Authors:** Gustav Magnusson, Maria Engström, Charalampos Georgiopoulos, Gunnar Cedersund, Lovisa Tobieson, Anders Tisell

**Affiliations:** ^1^Department of Health, Medicine and Caring Sciences, Linköping University, Linköping, Sweden; ^2^Center for Medical Image Science and Visualization (CMIV), Linköping University, Linköping, Sweden; ^3^Diagnostic Radiology, Department of Clinical Sciences, Medical Faculty, Lund University, Lund, Sweden; ^4^Department of Biomedical Engineering, Linköping University, Linköping, Sweden; ^5^Department of Neurosurgery in Linköping, and Department of Biomedical and Clinical Sciences, Linköping University, Linköping, Sweden

**Keywords:** cerebrovascular reactivity, CO_2_ gas challenge, ventilation, magnetic resonance imaging, carbon dioxide, vascular stimulus

## Abstract

**Introduction:**

Cerebrovascular reactivity imaging (CVR) is a diagnostic method for assessment of alterations in cerebral blood flow in response to a controlled vascular stimulus. The principal utility is the capacity to evaluate the cerebrovascular reserve, thereby elucidating autoregulatory functioning. In CVR, CO_2_ gas challenge is the most prevalent method, which elicits a vascular response by alterations in inspired CO_2_ concentrations. While several systems have been proposed in the literature, only a limited number have been devised to operate in tandem with mechanical ventilation, thus constraining the majority CVR investigations to spontaneously breathing individuals.

**Methods:**

We have developed a new method, denoted Additional CO_2_, designed to enable CO_2_ challenge in ventilators. The central idea is the introduction of an additional flow of highly concentrated CO_2_ into the respiratory circuit, as opposed to administration of the entire gas mixture from a reservoir. By monitoring the main respiratory gas flow emanating from the ventilator, the CO_2_ concentration in the inspired gas can be manipulated by adjusting the proportion of additional CO_2_. We evaluated the efficacy of this approach in (1) a ventilator coupled with a test lung and (2) in spontaneously breathing healthy subjects. The method was evaluated by assessment of the precision in attaining target inspired CO_2_ levels and examination of its performance within a magnetic resonance imaging environment.

**Results and discussion:**

Our investigations revealed that the Additional CO_2_ method consistently achieved a high degree of accuracy in reaching target inspired CO_2_ levels in both mechanical ventilation and spontaneous breathing. We anticipate that these findings will lay the groundwork for a broader implementation of CVR assessments in mechanically ventilated patients.

## 1 Introduction

Cerebrovascular reactivity imaging (CVR) represents an innovative approach for the non-invasive exploration of cerebral hemodynamics. It involves the application of a vasoactive stimulus and simultaneous measurements of alterations in cerebral blood flow. The reactivity, quantified as the change in blood flow divided by the applied stimulus, serves as an indirect indicator of the local vasoregulatory reserve within the cerebral vasculature. Furthermore, this method enables the computation of the time delay in the blood flow response. Research has extensively examined the application of the CVR technique across various medical conditions, including arterial stenosis, moyamoya disease, brain tumors, dementia, small vessel disease, and subarachnoid hemorrhage (1). Despite the promising clinical potential of CVR in these diverse patient cohorts, it has not yet achieved widespread clinical adoption and remains predominantly a research tool. One key impediment to its broader utilization is the limited availability of commercial products for stimulus generation that can be applied across different clinical scenarios.

The established vascular stimulus in CVR measurement is a controlled alteration of arterial carbon dioxide partial pressure (${\text{P}}_{{\text{CO}}_{2}}^{\text{a}}$). The associated changes in blood flow are typically monitored using magnetic resonance imaging (MRI) in conjunction with the blood oxygenation level dependent (BOLD) signal (2). Various methods can be employed to manipulate ${\text{P}}_{{\text{CO}}_{2}}^{\text{a}}$, such as controlled breathing patterns, including deep breathing and breath-holding, or the administration of vasoactive drugs like Acetazolamide (3). However, the preferred approach, due to its reliability and reproducibility, is the administration of carbon dioxide within the inspired gas (4). Several methods described in the literature use reservoirs with a variable mixture of CO_2_, O_2_, and N_2_, to target fractional CO_2_ concentrations in the inspired gas (${\text{F}}_{{\text{CO}}_{2}}^{\text{i}}$) (5, 6). More sophisticated methods incorporate advanced controls, such as dynamic end-tidal forcing or prospective end-tidal targeting, which enable precise targeting of subjects' end-tidal CO_2_/O_2_ levels, reflecting the gas concentrations in the alveoli (7, 8).

**Figure 1:**
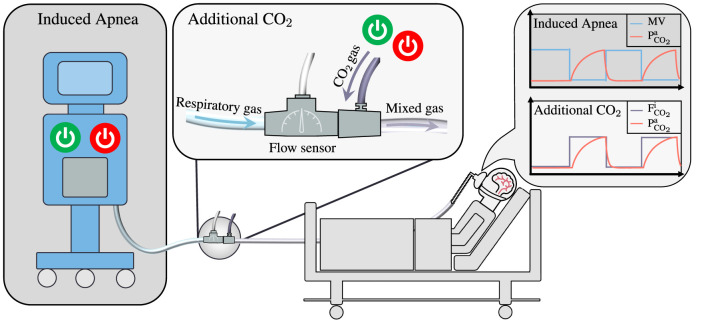
Illustrating the main differences between two methods employed for the measurement of Cerebrovascular Reactivity in mechanically ventilated patients: Induced Apnea and Additional CO_2_. The Induced Apnea method, commonly referred to as “breath-hold”, produces a hypercapnic stimulus by temporary switching off the ventilator, resulting in a transient cessation of the patient's minute ventilation (MV). This leads to an increase in arterial CO_2_ levels (${\text{P}}_{{\text{CO}}_{2}}^{\text{a}}$), which subsequently revert to baseline upon reactivation of the ventilator. This method has been illustrated by Fierstra et al. (12). In contrast, the Additional CO_2_ method maintains continuous ventilation as the ventilator operates without interruption. Instead, it introduces high-concentration CO_2_ intermittently into the breathing circuit, modulating the composition of inspired gases. The flow of respiratory gas is continuously measured through a flow sensor, while a mass flow controller (not shown) regulates the admixture of CO_2_ to maintain a predetermined target CO_2_ concentration in the inspired gas (${\text{F}}_{{\text{CO}}_{2}}^{\text{i}}$). Given the uninterrupted operation of the ventilator, continuous monitoring of the patient's O_2_ and CO_2_ levels ensues, offering an added layer of safety and control.

While the literature contains substantial information on methods for CO_2_ gas challenge in spontaneously breathing patients, there has been limited exploration in mechanically ventilated patients (9, 10). This gap in research may explain why CVR studies in mechanically ventilated patients have primarily focused on breathing pattern alterations or administration of vasoactive drugs (11–14). The aim of this study is to implement a method capable of administering CO_2_ to both ventilated and non-ventilated patients. In contrast to other CO_2_ administration methods, our method does not generate the entire gas mixture but, instead, supplements the inspired gas with additional CO_2_ in proportion to the respiratory gas flow, as illustrated in Figure 1, drawing inspiration from nitric oxide systems (15). This approach does therefore not rely on the addition of external reservoirs or multiple gas sources, keeping the necessary modification of the breathing circuit minimal, which is desirable in an intensive care setting. We conducted tests of our method within mechanical ventilation, using a test lung, and in spontaneously breathing healthy subjects, comparing it to a conventional CO_2_ gas challenge method, inspired by the approach detailed by Tancredi et al. (5).

## 2 Materials and methods

In this section, we outline the material and equipment employed in evaluating the Additional CO_2_ method, directing readers to the Supplementary material for a comprehensive description of specific components used.

### 2.1 Rationale

While conducting CVR experiments, the manipulation of gases arterial partial pressures is common practice, yet direct measurement of these pressures is infrequently performed. Instead, alveolar pressures, derived from end-tidal values, are typically used due to their strong correlation with arterial pressures in lung-healthy individuals (16). In the alveolar, we typically talk about gases fractional concentrations (${\text{F}}_{\text{gas}}^{\text{A}}$) and partial pressures (${\text{P}}_{\text{gas}}^{\text{A}}$) interchangeably since there is a direct translation between the two: ${\text{P}}_{\text{gas}}^{\text{A}}={\text{F}}_{\text{gas}}^{\text{A}}\times \left({\text{P}}_{\text{atm}}-{\text{P}}_{{\text{H}}_{2}\text{O}}\right)$, where P_atm_ is the atmospheric pressure and P_H_2_O_ is the partial pressure of water vapor at body temperature. The steady-state fractional alveolar concentrations of O_2_ and CO_2_ (${\text{F}}_{{\text{O}}_{2}/{\text{CO}}_{2}}^{\text{A}}$) are determined by alveolar ventilation (${\dot{\text{V}}}_{\text{A}}$), O_2_ consumption/CO_2_ production (${\dot{\text{V}}}_{{\text{O}}_{2}/{\text{CO}}_{2}}$), and inspired gas concentrations (${\text{F}}_{{\text{O}}_{2}/{\text{CO}}_{2}}^{\text{i}}$), as described by Equations (1, 2) (8).


(1) $$\begin{array}{ll} {\text{F}}_{{\text{O}}_{2}}^{\text{A}} & ={\text{F}}_{{\text{O}}_{2}}^{\text{i}}-\frac{{\dot{\text{V}}}_{{\text{O}}_{2}}}{{\dot{\text{V}}}_{\text{A}}} \end{array}$$

(2) $$\begin{array}{ll} {\text{F}}_{{\text{CO}}_{2}}^{\text{A}} & ={\text{F}}_{{\text{CO}}_{2}}^{\text{i}}+\frac{{\dot{\text{V}}}_{{\text{CO}}_{2}}}{{\dot{\text{V}}}_{\text{A}}} \end{array}$$

Equation (2) elucidates potential manipulation of ${\text{F}}_{{\text{CO}}_{2}}^{\text{A}}$, with controlled breathing patterns influencing alveolar ventilation ${\dot{\text{V}}}_{\text{A}}$, administration of vasoactive drugs altering ${\dot{\text{V}}}_{{\text{CO}}_{2}}$, or direct modification inspired CO_2_. Among these techniques, direct manipulation of inspired CO_2_ offers increased repeatability, as it targets the corresponding parameter in Equation (2) directly.

A common and straightforward method to modify inspired CO_2_ is to adjust the gas content in a reservoir from which the subject breathes. However, in mechanical ventilation, this presents challenges due to the presence of internal one-way valves in the ventilator, ensuring gas flow in only one direction. One workaround is illustrated by Winter et al. (9), involving the placement of two external reservoirs in a sealed compartment. Gas control occurs in one reservoir from which the subject breathes, while the ventilator ventilates the other. However, this approach necessitates a complex external breathing circuit and requires patient separation from the ventilator. Another option is manual ventilation, where gas control in a self-inflated bag occurs simultaneously with manual compression. Nonetheless, this method does not equate to mechanical ventilation (10). These limitations of reservoir-based alterations in mechanical ventilation may explain why most CVR studies on ventilated patients primarily focus on either breathing pattern alterations or administration of vasoactive drugs (11–14). Ideally, a simple method enabling ${\text{F}}_{{\text{CO}}_{2}}^{\text{i}}$ alterations while requiring minimal modification of the breathing circuit and no separation of the ventilator and patient would be preferable.

The proposed Additional CO_2_ method addresses this need. By measuring respiratory gas flow and adding a proportional flow of CO_2_, it does not rely on a reservoir to alter the gas content in inspired gas. The only modification to the breathing circuit is the incorporation of a flow sensor, gas inlet, and potentially a humidifier (if not already included) to facilitate mixing of the additional CO_2_ and respiratory gas. Moreover, since the method relies on the measurement of respiratory gas flow, it has the potential to be further developed to enable direct targeting of ${\text{F}}_{{\text{CO}}_{2}}^{\text{A}}$ by adjusting the administered ${\text{F}}_{{\text{CO}}_{2}}^{\text{i}}$ on a breath-to-breath basis according to changes in ${\dot{\text{V}}}_{\text{A}}$ (see Equation 2). Additionally, a source of oxygen could also be incorporated to allow simultaneous targeting of ${\text{F}}_{{\text{O}}_{2}}^{\text{A}}$.

### 2.2 Additional CO_2_ system

A prototype system, Additional CO_2_ System, was devised to assess the Additional CO_2_ method, consisting of four primary components: gas source (100 % CO_2_, 5 L canister, AirLiquide), a control unit (including a microcontroller, Arduino Beetle, DFRobotic), gas control (flow sensor, SFM3200, Sensirion and mass flow controller, SFC5500, Sensirion) and graphical user interface (GUI, Python program running on a laptop, in-house developed). The flow sensor was read by the control unit, which also managed the mass flow controller. The proportional relationship between the setpoint of the mass flow controller and the flow of the flow sensor was computed at the GUI and transmitted to the control unit. The underlying calculation involved the solving of a mass balance equation for a target ${\text{F}}_{{\text{CO}}_{2}}^{\text{i}}$:


(3) $$\begin{array}{l} {\text{ F}}_{{\text{CO}}_{2}}^{\text{i}}=\frac{{\text{F}}_{{\text{CO}}_{2}}^{\text{res}}\times {\dot{\text{M}}}_{\text{res}}+{\text{F}}_{{\text{CO}}_{2}}^{\text{add}}\times {\dot{\text{M}}}_{\text{add}}}{{\dot{\text{M}}}_{\text{res}}+{\dot{\text{M}}}_{\text{add}}}\\ \Rightarrow \frac{{\dot{\text{M}}}_{\text{add}}}{{\dot{\text{M}}}_{\text{res}}}=\frac{{\text{F}}_{{\text{CO}}_{2}}^{\text{i}}-{\text{F}}_{{\text{CO}}_{2}}^{\text{res}}}{{\text{F}}_{{\text{CO}}_{2}}^{\text{add}}-{\text{F}}_{{\text{CO}}_{2}}^{\text{i}}} \end{array}$$

where ${\dot{\text{M}}}_{\text{res/add}}$ and ${\text{F}}_{{\text{CO}}_{2}}^{\text{res/add}}$ are the mass flow and fractional CO_2_ concentration of the respiratory (res) and additional (add) gas. A consequence of introducing additional CO_2_ in this manner is the concurrent reduction of O_2_ concentration in the inspired gas. The GUI also displayed this change in ${\text{F}}_{{\text{O}}_{2}}^{\text{i}}$ to the user. Moreover, to ensure safety, strict limits were imposed on the maximal and minimal ${\text{F}}_{{\text{CO}}_{2}}^{\text{i}}$ and ${\text{F}}_{{\text{O}}_{2}}^{\text{i}}$ concentrations, set at 5 and 19 %, respectively.

To specify the target ${\text{F}}_{{\text{CO}}_{2}}^{\text{i}}$ concentration, a user loaded a JSON protocol file containing target values and corresponding time durations via the GUI. For a more comprehensive description of the constructed system and the components employed, refer to Section 1.1 in the Supplementary material.

### 2.3 Reservoir CO_2_ system

A reference system, modeled after the design by Tancredi et al. (5), was assembled to facilitate a comparative analysis with our Additional CO_2_ System in spontaneous breathing. This system, from here on referred to as Reservoir CO_2_ System, was established using three mass flow controllers (SLA5850, Brooks Instrument) connected to sources of O_2_, CO_2_, and N_2_. By altering the setpoints of each controller, a specific gas mixture was created and stored in a reservoir, from which a subject would draw breath. The same GUI mentioned earlier was employed to oversee the mass flow controllers. Users could specify target ${\text{F}}_{{\text{O}}_{2}}^{\text{i}}$ and total flow rate, in addition to ${\text{F}}_{{\text{CO}}_{2}}^{\text{i}}$ concentrations, using a protocol file similar to the one used for the Additional CO_2_ System. The same constraints on maximal and minimal ${\text{F}}_{{\text{CO}}_{2}}^{\text{i}}$ and ${\text{F}}_{{\text{O}}_{2}}^{\text{i}}$ concentrations, as described above, remained in effect.

Note that this system was exclusively used during in spontaneous breathing and not during in mechanical ventilation (see Section 3.1 below). The reason being that the one-way valves within a mechanical ventilator only allow gas in the inspiration part of the circuit to flow toward the patient. Without more sophisticated modification to the breathing circuit, as described by Winter et al. (9) or Venkatraghavan et al. (10), control over the gas in a breathing reservoir is not achievable. For further details regarding the system and its components, please refer to Section 1.2 in the Supplementary material.

### 2.4 Ventilator and test lung

To evaluate the Additional CO_2_ System in conjunction with mechanical ventilation, an anesthesia workstation (Primus Infinity Empowered, Dräger Medical) was used together with a test lung (AccuLung, Fluke Biomedical). The workstation was also used for sampling inspired and expired O_2_ and CO_2_.

### 2.5 Breathing circuits

**Figure 2:**
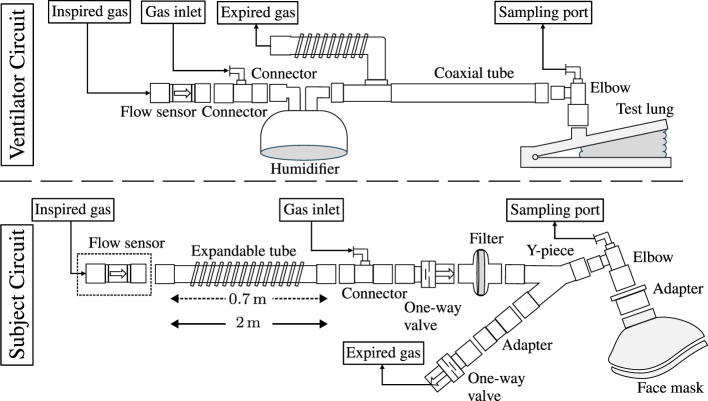
A schematic representation of the respiratory circuits employed in mechanical ventilation of a test lung (Ventilator Circuit) and spontaneously breathing healthy subjects (Subject Circuit). In the Ventilator Circuit, the respiratory circuit encompasses a flow sensor affixed to the outlet of the ventilator (not depicted), followed by a connector equipped with a luer port to facilitate the introduction of additional CO_2_. To ensure the homogeneity of the gas mixture, an empty humidifier was incorporated to enable gas mixing. A coaxial tube connected to the humidifier and to the ventilator's inlet, with the distal end attached to the test lung via an elbow featuring a sampling port. In the Subject Circuit, the configuration of the respiratory circuit differed slightly for the Additional CO_2_ and Reservoir CO_2_ Systems. In the Reservoir CO_2_ configuration, the deployment of a flow sensor was omitted, and the extendable tube was elongated from its minimal length of 0.7 m, as used in the Additional CO_2_ configuration, to a length of 2 m, serving as a reservoir for the administered gas. The expandable tube was affixed to the gas inlet connector, followed by one-way valves and a Y-connector separating the inspiration and expiration part of the circuit. A filter prevented particles from reaching the subjects who were breathing in the circuit through a face mask, which was fitted to the head with the help of an adjustable harness (not shown).

Two distinct breathing circuits were employed: one for mechanical ventilation of a test lung (Ventilator Circuit) and another for spontaneously breathing healthy subjects (Subject Circuit), as illustrated in Figure 2. In the Ventilator Circuit, which was only used together with the Additional CO_2_ System, the flow sensor was connected to the ventilator's outlet, followed by a connector with a gas inlet to which the mass flow controller's outlet was attached. An empty humidifier was positioned immediately after the connector to serve as a small volume, ensuring a uniform gas mixture. A coaxial ventilator tube was affixed to the outlet of the humidifier, and an elbow connector with a sampling port connected the tube to the test lung.

In the Subject Circuit, the lower part of Figure 2 depicts the configurations used for the Additional CO_2_ and Reservoir CO_2_ Systems. The sole differences between the Additional CO_2_ and Reservoir CO_2_ configurations were the inclusion of the flow sensor (used solely in the Additional CO_2_ System) and the length of the expandable tube. In the Reservoir CO_2_ System, the expandable tube functioned as the gas reservoir, as elucidated by Tancredi et al. (5), and was extended to a length of 2 m, creating a reservoir with a size of about 700 ml (tube diameter ~22 mm). Given that normal tidal volumes in adults are ~500 ml, this size was deemed sufficient (17). Conversely, for the Additional CO_2_ System, the expandable tube was minimized to 0.7 m. The reason for not completely removing the tube was the desire to maintain the flow sensor away from the center of the MRI scanner to avoid interference when using the system in a full BOLD-CVR setup (see Section 3.2 below). Apart from these variances, the breathing circuit remained uniform for the Additional CO_2_ and Reservoir CO_2_ Systems and comprised a connector with a gas inlet for the addition of pure CO_2_ gas (in the Additional CO_2_ System) or a gas mixture of O_2_, CO_2_, and N_2_ (in the Reservoir CO_2_ System). The direction of gas flow was regulated by two one-way valves, and a filter was added to eliminate particles from the inspired gas. A Y-piece separated the inspiration and expiration segments of the circuit, with an elbow connector featuring a sampling port connecting the Y-piece to the face mask (Mask 7450 V2, Vyaire). An in-house 3D printed adapter was used to accommodate the 22 mm elbow to the 30 mm port of the face mask. For a comprehensive inventory of components used, please refer to Supplementary Table S1.

## 3 Method

### 3.1 Assessment of inspired CO_2_ target accuracy

The primary objective of this study was to assess the accuracy of the Additional CO_2_ method in achieving the desired CO_2_ target levels within the inspired gas. This assessment was conducted under two distinct scenarios: mechanical ventilation and spontaneous breathing.

**Figure 3:**
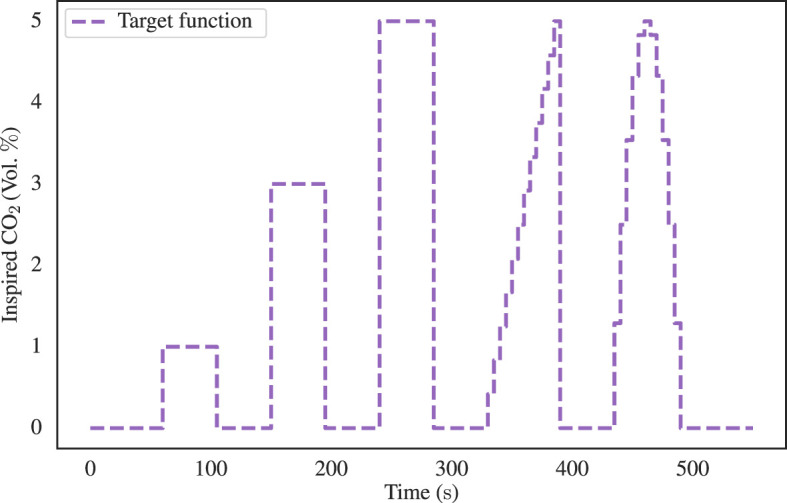
The inspired CO_2_ target function employed for the assessment of the precision of the proposed Additional CO_2_ method. The target function comprises three 45 s box-stimulus intervals, each at distinct CO_2_ concentrations of 1%, 3%, and 5%. These stimuli were subsequently followed by a 60 s ramp and half sinusoidal waveform, both characterized by a peak concentration of 5% CO_2_. A 45 s baseline was inserted between each stimulus and an initial and final baseline of 60 s duration was also included.

The ${\text{F}}_{{\text{CO}}_{2}}^{\text{i}}$ target function employed in this evaluation encompassed a range of stimulus types, as illustrated in Figure 3. These stimuli included three box-stimulus at 1%, 3%, 5% CO_2_, each lasting for 45 s, with an initial 60 s baseline period and a 45 s intermediate baseline. Subsequently, a ramp function was applied, increasing CO_2_ concentration from 0 to 5% over 60 s, followed by the first half of a sinusoidal waveform with a peak concentration of 5 % and a time period of 120 s. Finally, a 60 s baseline was appended, resulting in a total protocol duration of ~9 min.

#### 3.1.1 Inspired CO_2_ target accuracy in mechanical ventilation

The accuracy of inspired CO_2_ levels during mechanical ventilation was evaluated using an anesthesia workstation (Primus Infinity Empowered, Dräger Medical) in conjunction with a test lung (AccuLung, Fluke Biomedical), and the Ventilator Circuit shown in the top part of Figure 2. To ensure a comprehensive assessment, a variety of ventilator conditions were considered, aligning with the specifications established by the European standard ISO 80601-2-12:2020 (18). Within this standard, two specific categories were explored: volume-control inflation (table 201.104) and pressure-control inflation (table 201.105). Due to limitations in the available settings of the AccuLung test lung, only the initial seven test cases from each table, totaling 14 test cases, were feasible. The complete list of these test cases is provided in Supplementary Table S4.

For each test case, randomized in order, the Additional CO_2_ System administered CO_2_ according to the ${\text{F}}_{{\text{CO}}_{2}}^{\text{i}}$ target function depicted in Figure 3 and the mass balance equation shown in Equation (3). The Primus workstation, functioning both as a ventilator and gas analyzer, continuously sampled O_2_ and CO_2_ concentrations through the sampling port of the breathing circuit at an approximate frequency of 60 Hz. Using the sampled O_2_ and CO_2_ curves, the inspired O_2_ and CO_2_ levels were calculated by an automated Python script. The script identified the inspiratory phase and computed both the peak and baseline levels of O_2_ and CO_2_ to measure the variability within each inspiration. These values were subsequently interpolated to ensure uniform sampling across all 14 ventilator test cases which enabled aggregation and computation of mean plus confidence intervals using the built-in functionalities of the Seaborn package in Python.

To effectively compare the aggregated values with the target ${\text{F}}_{{\text{O}}_{2}}^{\text{i}}$ and ${\text{F}}_{{\text{CO}}_{2}}^{\text{i}}$ levels, the aggregated data was time shifted 8 s to compensate for the sampling delay of 3 s and the presence of dead space within the ventilator tubing. This dead space necessitated multiple breaths before any alteration in inspired CO_2_ concentrations would manifest at the sampling port. While it is acknowledged that individual test runs would have experienced distinct time delays, accounting for variations in tidal volume and respiratory rate, it was determined that the uniform application of the same delay to all runs introduced a relatively minor error when compared with other sources of variation, such as the temporal misalignment between the onset of the stimulus and the start of the subsequent breath.

#### 3.1.2 Inspired CO_2_ target accuracy in spontaneous breathing

To conduct a comprehensive evaluation of the Additional CO_2_ method, six healthy subjects (aged between 25 and 42, three males and three females) were recruited to assess accuracy of inspired CO_2_ in spontaneous breathing. Additionally, we made a comparative analysis between our proposed system: Section 2.2, and the previously described system outlined by Tancredi et al. (5): Section 2.3.

The recruitment process strictly adhered to the principles outlined in the Helsinki Declaration, and ethical approval was obtained from the Swedish Ethical Review Authority (reference number: 2021-04825). Prior to their participation, the selected subjects underwent a screening process to ascertain the absence of pulmonary diseases or other chronic health conditions.

The Subject Circuit, lower part of Figure 2, was used in its different configurations for the Additional CO_2_ and Reservoir CO_2_ Systems. The acquisition of O_2_ and CO_2_ concentrations was made by the Primus workstation, now operating in surveillance mode. Notably, the workstation was not connected to the inspiration and expiration portions of the Subject Circuit, as these remained open to the surrounding room environment.

The same ${\text{F}}_{{\text{CO}}_{2}}^{\text{i}}$ target function employed in the mechanical ventilation assessment was used (see Figure 3). Subjects were instructed to maintain calm and normal breathing while the target stimulus was administered. The experiment was repeated for both the Additional CO_2_ and Reservoir CO_2_ configurations for each subject, resulting in a total of 12 experimental runs. The sequence in which these two methods were employed was randomized in blocks to mitigate any order effects. Furthermore, it is relevant to mention that the Reservoir CO_2_ method allowed for the specification of the total flow of fresh gas and inspired O_2_ levels, which is not actively controlled in the Additional CO_2_ method. To facilitate comparison between the two methods, the target ${\text{F}}_{{\text{O}}_{2}}^{\text{i}}$ level in the Reservoir CO_2_ System was set to 21 %, approximately corresponding to the ambient room concentration. Achieving the 15 L/min flow rate of fresh gas, as proposed by Tancredi et al. (5), was unattainable due to constraints imposed by the maximum flow capacity of the mass flow controllers in the Reservoir CO_2_ System, which was limited to 10 L/min. Instead, we chose to use 8 L/min of fresh gas flow, which is approximately the upper limit of common minute ventilation in healthy adults which range between 6 and 8 L/min (17, 19). This also means minimum excessive usage of fresh gas, making the method comparable with the Additional CO_2_ method, where gas is never delivered in excess but always in proportion to the measured respiratory gas flow.

A semi-automated Python script was used to compute inspired and end-tidal O_2_ and CO_2_ values from the sampled concentrations. Manual intervention was primarily required because of the interplay between inspired CO_2_ and subject production of CO_2_ during spontaneous breathing. Consequently, the sampled CO_2_ trace exhibited increased variability, multiple peaks, and valleys compared to the more stable curves observed in passive ventilated test lung scenarios. The semi-automated script initially sought to identify expiration segments within the O_2_/CO_2_ traces by leveraging the characteristic exponential decay pattern typically exhibited during expiration. Subsequently, these expiration phases were used to pinpoint inspiration phases, situated between two expiration phases. To fully capture the range of values within each inspiration, 1–3 data points were tracked. Inspection of the script's initial estimations, including manual refinements when needed, was then performed. The script achieved a success rate of ~95 % in accurately identifying expiration/inspiration phases. In the case of the Reservoir CO_2_ System, the non-excessive flow of fresh gas (as discussed above) meant that primarily the initial inspired gas was being targeted well, with noticeable reduction in CO_2_ levels during the late inspiration phase. However, since the late inspired gas typically does not reach the alveolar space but remains within the physiological dead-space, it was deemed motivated to disregard these late reductions in inspired CO_2_ levels to avoid potentially biasing the accuracy of the Reservoir CO_2_ System negatively. While this approach may introduce a positive bias, it was considered preferable over a negative bias, particularly since the Reservoir CO_2_ System served as the reference system for the Additional CO_2_ System.

Data aggregation across subjects was facilitated by binning the identified inspired and end-tidal O_2_ and CO_2_ values into 5 s bins, a process that also introduced some degree of smoothing. Consequently, the need for time-shifting to compensate for the sampling delay of 3 s was eliminated. Inspired values from individual runs could then be aggregated across all subjects and directly compared to the target ${\text{F}}_{{\text{O}}_{2}}^{\text{i}}$ and ${\text{F}}_{{\text{CO}}_{2}}^{\text{i}}$ levels, again with the help of Seaborn package in Python. For the end-tidal O_2_ and CO_2_ values, baseline subject variations were first removed by subtracting the mean end-tidal value during the initial 60 s of each run before aggregating across subjects.

### 3.2 BOLD-CVR examination

In the context of assessing the Additional CO_2_ method as a technique for CVR measurement, it is essential to note that the conventional approach to conducting CVR examinations relies on using the BOLD signal as a surrogate measure of blood flow. We therefore evaluated the Additional CO_2_ method in an MRI environment in a subset of two subjects. They underwent BOLD-CVR examinations using both the Additional CO_2_ and Reservoir CO_2_ Systems, which were repeated twice in a test-retest experimental design, yielding a total of four runs per subject. The same Subject Circuit from the target accuracy assessment (lower part of Figure 2) was again used. Detailed information regarding the experiment, including MRI and CO_2_ protocols, as well as the generation of CVR maps, can be found in Section 2 of the Supplementary material.

Note that the Additional CO_2_ System is also safe to use together with the Ventilator Circuit (upper part of Figure 2) inside an MRI environment, of course an MR Conditional ventilator will have to be used.

## 4 Results

### 4.1 Target accuracy of inspired gases

**Figure 4:**
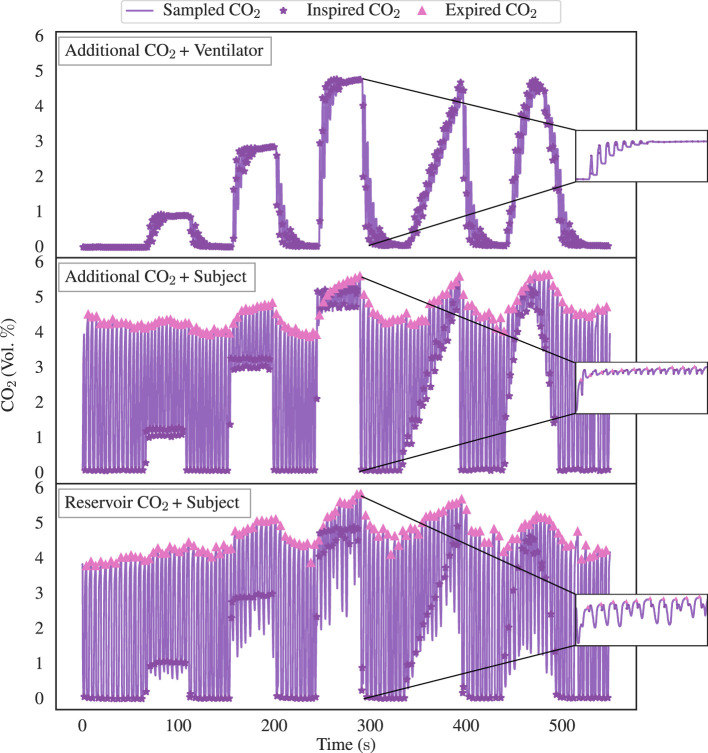
Illustrative data pertaining to inspired CO_2_ target accuracy assessment, delineating the three distinct experimental configurations: the Additional CO_2_ System + Ventilator Circuit (depicted in the top graph), the Additional CO_2_ System + Subject Circuit (displayed in the middle graph), and the Reservoir CO_2_ System + Subject Circuit (depicted in the bottom graph). All three instances use the identical target function as showcased in Figure 3. Upon closer examination within the magnified windows, we see the dynamic fluctuations in CO_2_ concentration throughout the 5 % box-stimulus. It becomes evident that the rise time of CO_2_ in the Ventilator Circuit exhibits a substantially slower response in comparison to the other two configurations. Furthermore, note the greater degree of variability in inspired CO_2_ levels for the Reservoir CO_2_ System when compared with the Additional CO_2_ System. This is due to the non-excessive usage of fresh gas in our Reservoir CO_2_ System, leading to a reduced inspired concentration at the end of the inspiration phase. However, for the sake of inspired CO_2_ target accuracy of the Reservoir CO_2_ System, only the concentration during the initial inspired phase is being tracked.

The present section delves into the outcomes of the experiment aimed at evaluating the target accuracy of inspired CO_2_. To illustrate the analysis, Figure 4 shows example datasets. In the uppermost section of Figure 4, the Additional CO_2_ System with the Ventilator Circuit is depicted, along with a randomly selected test case. The middle section showcases the Additional CO_2_ System with the Subject Circuit in conjunction with a random subject, while the lowermost part illustrates the Reservoir CO_2_ System with the same subject. In all instances, the same ${\text{F}}_{{\text{CO}}_{2}}^{\text{i}}$ target function, as detailed in Figure 3, was used. Focusing on the magnified windows, the dynamic fluctuations in CO_2_ concentration throughout the 5% box-stimulus is shown. We see that the rise time of CO_2_ in the Ventilator Circuit is longer than the other two configurations. Also, the Reservoir CO_2_ System has a greater degree of variability in inspired CO_2_ levels when contrasted with the Additional CO_2_ System. This is due to the non-excessive usage of fresh gas in the Reservoir CO_2_ System, resulting in a sudden reduction of inspired CO_2_ concentration at the end of the inspiration phase. To avoid negatively biasing the inspired CO_2_ target accuracy for the Reservoir CO_2_ System, only the initial phase of the inspiration is being tracked, as discussed in Section 3.1.2.

**Figure 5:**
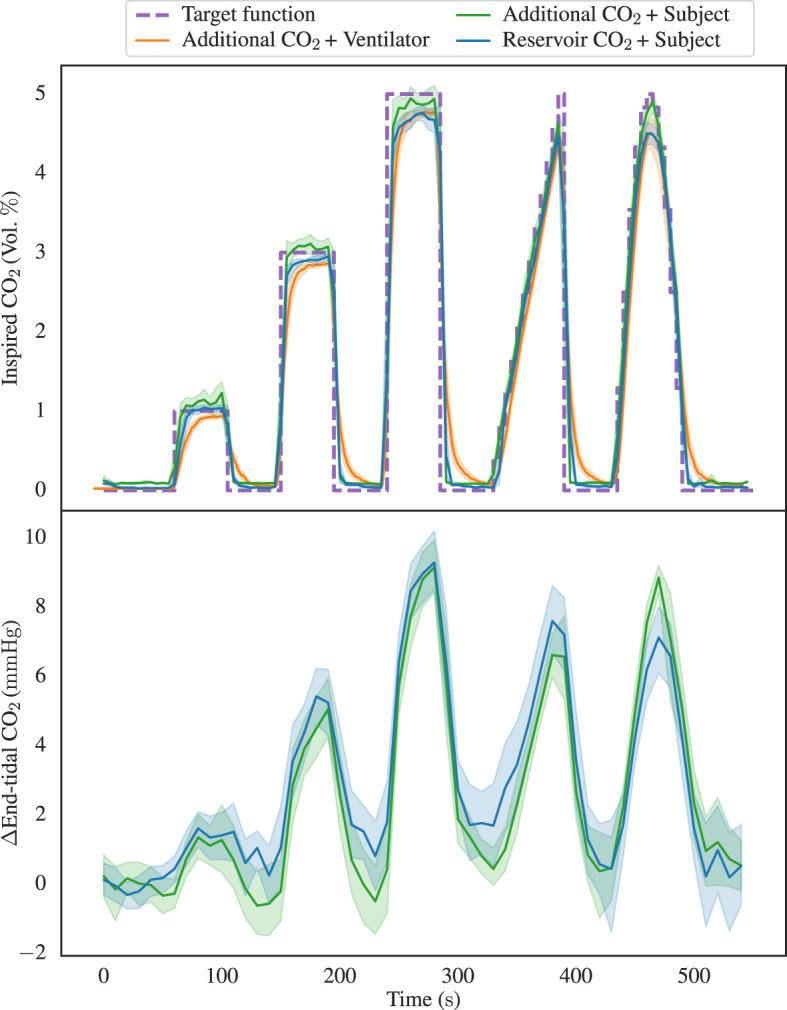
Illustrating the aggregated inspired CO_2_ concentrations in the top graph with data from the three distinct configurations: Additional CO_2_ System + Ventilator Circuit, Additional CO_2_ System + Subject Circuit, and Reservoir CO_2_ System + Subject Circuit. The data is depicted in terms of both the mean values and a 95% confidence interval, alongside the inspired CO_2_ target function. Notably, it becomes evident that the Additional CO_2_ System performs equally good as the Reservoir CO_2_ System in its ability to attain diverse CO_2_ levels, although a consistent undershoot is observed in the ventilator configuration. In the lower graph, the aggregated end-tidal CO_2_ baseline deviations from the two sets of subject data are presented. We here again notice the similarity between the two methods.

To ascertain the performance of the experimental configurations the data from all runs were aggregated over all test cases/subjects as outlined in the Section 2. This process has enabled the computation of the mean and a 95 % confidence interval for the inspired/end-tidal CO_2_, as displayed in Figure 5. Significantly, it is apparent that the Additional CO_2_ System achieves a similar inspired CO_2_ target accuracy as the Reservoir CO_2_ System, albeit with a consistent undershoot noted in the Ventilator Circuit. Also, similar end-tidal CO_2_ changes are seen in both systems. Note that end-tidal values have been converted from volume percentages to partial pressures, assuming an atmospheric pressure of 760 mmHg and water vapor partial pressure of 47 mmHg.

**Figure 6:**
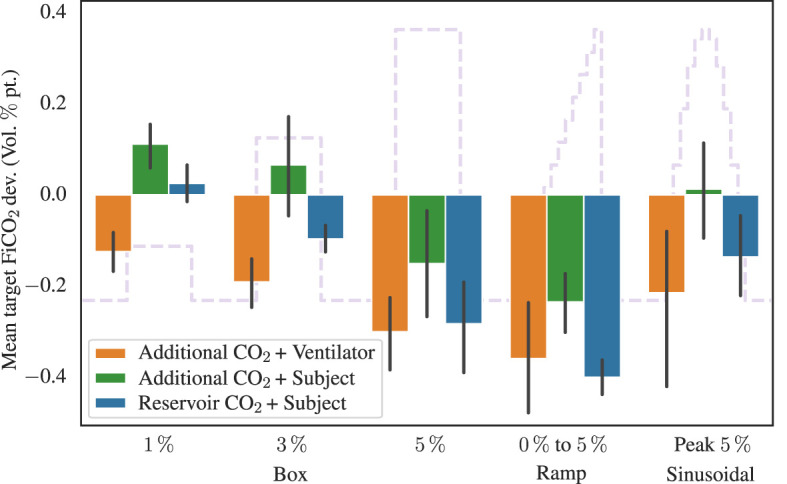
Showing the mean inspired CO_2_ target deviation, in volume percentage point, for the different stimuli and experimental configurations. The target function from Figure 3 is shown in the background. Transition periods for the box-stimuli (initial 10 s and final 5 s) and ramp-stimulus (final 5 s), have been removed before calculating the mean deviation. Also shown are 95%-confidence interval error bars.

The accuracy and precision of each setup, assessed by the mean deviation between measured and target ${\text{F}}_{{\text{CO}}_{2}}^{\text{i}}$, was quantified for each type of stimulus. The deviations, after eliminating transition periods for the box-stimuli (initial 10 s and final 5 s) and ramp-stimulus (final 5 s), are summarized in Figure 6. We see that the Additional CO_2_ System is consistently targeting the inspired CO_2_ to an accuracy better than 0.4 percentage points and performs similar well as the Reservoir CO_2_ System across all targets.

**Figure 7:**
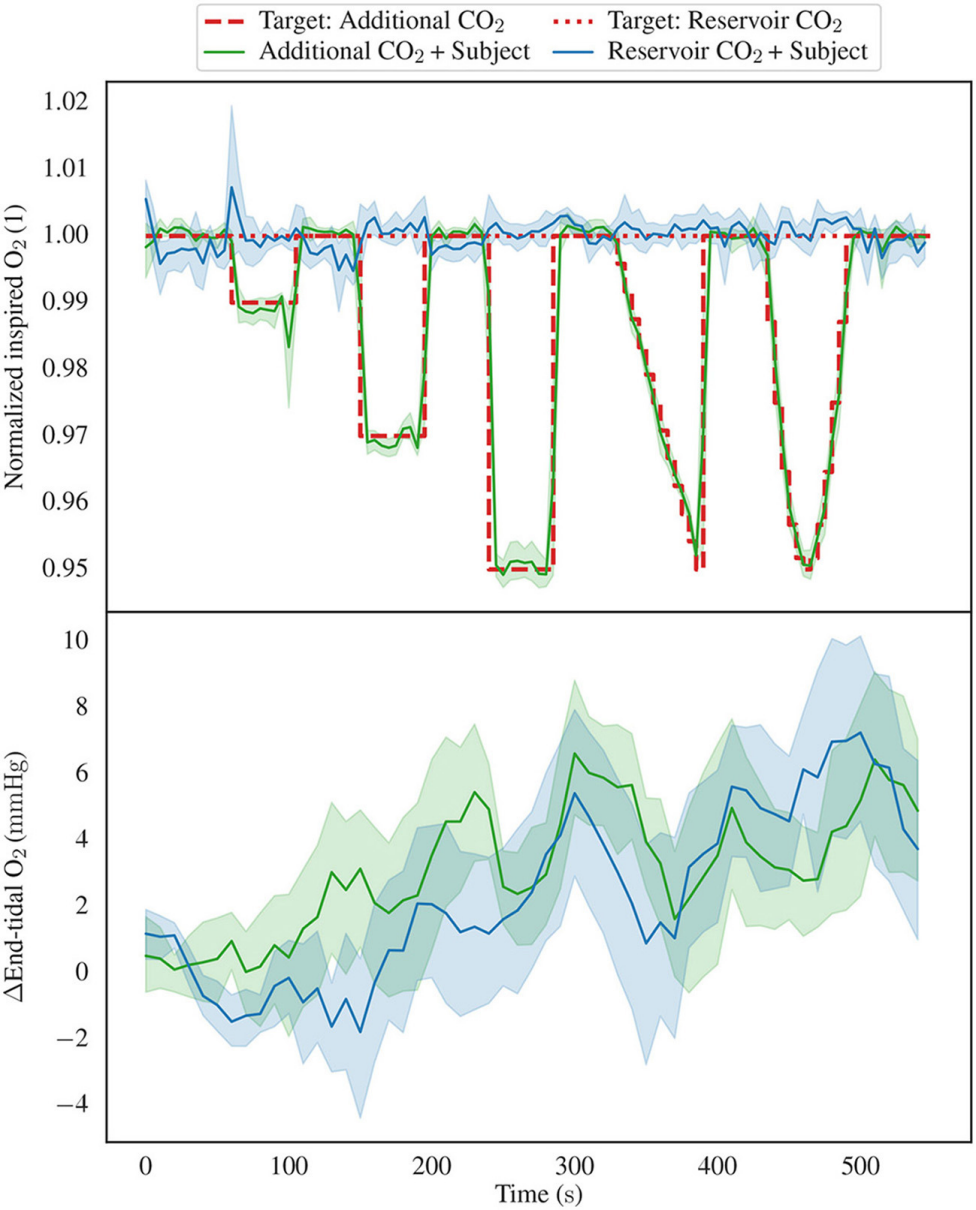
Showing the inspired and end-tidal O_2_ levels, focusing exclusively on the Additional CO_2_ System + Subject Circuit and Reservoir CO_2_ System + Subject Circuit configurations. The top graph displays the inspired O_2_, including both the mean values and 95% confidence intervals. It is noteworthy that two distinct target function are depicted, in the Reservoir CO_2_ configuration, the target inspired O_2_ concentration remains constant, while in the Additional CO_2_ configuration, it varies due to the introduction of additional CO_2_. Further, the measured and target inspired CO_2_ concentrations have been normalized by their baseline value to allow for direct comparison between the two methods. The lower graph presents the aggregated end-tidal O_2_ baseline deviations. Notably, both graphs exhibit analogous trends characterized by an increase in end-tidal O_2_ levels over time, despite the notable disparity in inspired O_2_ concentrations between the two experimental configurations.

Next, we redirect our attention toward the aggregated values of inspired and end-tidal oxygen, as depicted in Figure 7, with the uppermost graph showing the inspired O_2_ levels, and the lower graph showcasing the end-tidal O_2_ values. We restrict us to presentation of data from the Additional CO_2_ System + Subject Circuit and Reservoir CO_2_ System + Subject Circuit configurations. Even though both systems target the same baseline O_2_ concentration, 21 %, the Additional CO_2_ System did so passively by the usage of room air, which is not exactly 21 %. To facilitate direct comparison between the two configurations, the measured and target ${\text{F}}_{{\text{O}}_{2}}^{\text{i}}$ levels have been normalized (divided) by their baseline value in Figure 7. Notably, even though the inspired O_2_ varies drastically between the two methods, the end-tidal O_2_ demonstrate similar patterns marked by a successive rise over time.

### 4.2 BOLD-CVR measurements

**Figure 8:**
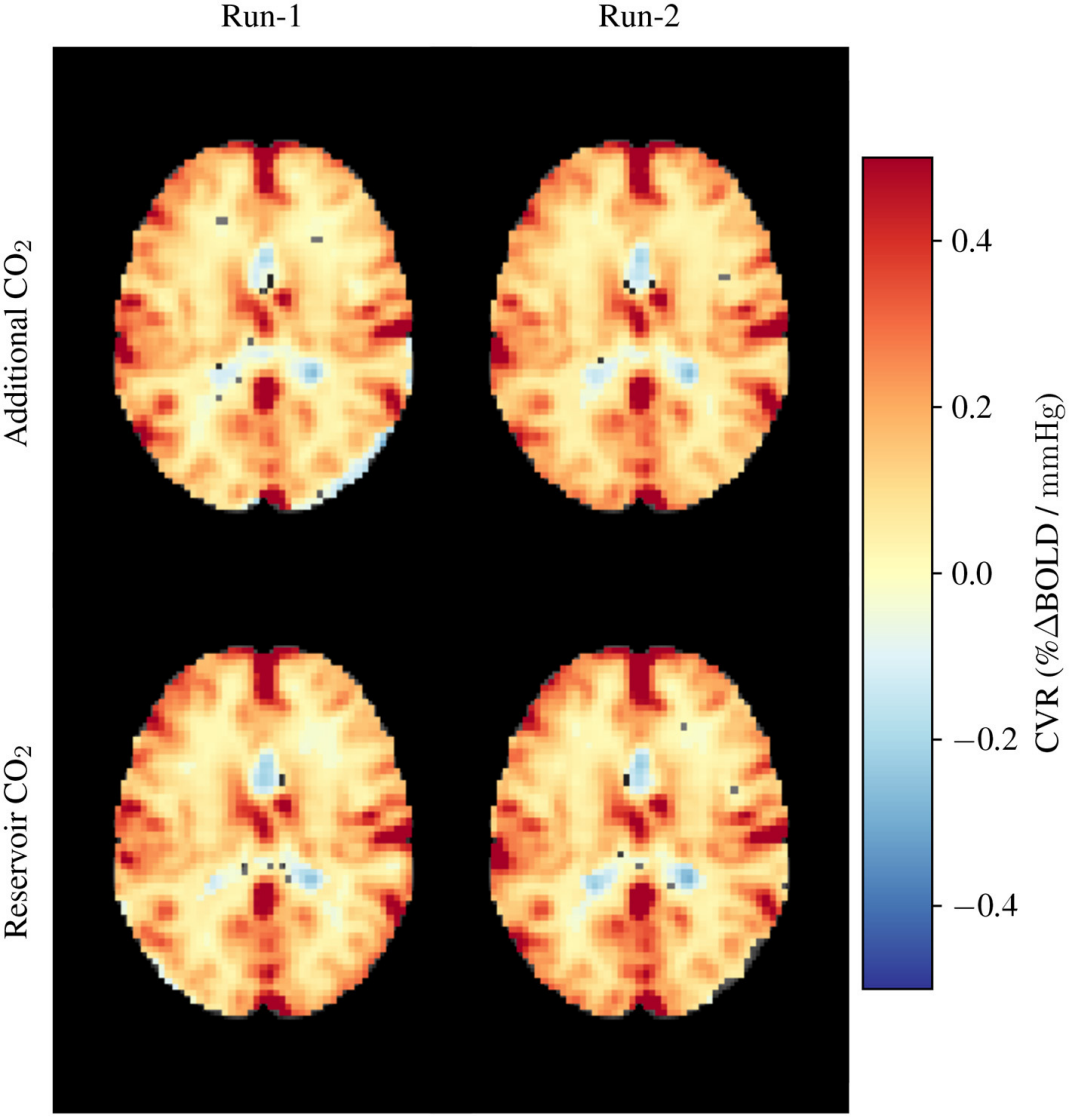
Exemplar CVR maps obtained from a single participant. The upper row showcases CVR maps generated using the Additional CO_2_ System, while the lower row exhibits CVR maps derived from the Reservoir CO_2_ System. These measurements were replicated twice for each system configuration.

In Figure 8, we present illustrative BOLD-CVR maps obtained through the application of the Additional CO_2_ and Reservoir CO_2_ Systems within a single subject. It is imperative to emphasize that our objective is not to derive quantitative inferences, however, Figure 8 does unveil a qualitative congruence in the CVR maps yielded by both methods.

## 5 Discussion

### 5.1 Inspired CO_2_ target attainment

When examining the illustrative data presented in Figure 4, noticeable disparities among the three distinct configurations (Additional CO_2_ System + Ventilator Circuit, Additional CO_2_ System + Subject Circuit, and Reservoir CO_2_ System + Subject Circuit) become evident. First, the ventilator configuration displays slower CO_2_ response compared to the two subject configurations. The distinct behavior arises primarily from the gas inlet's placement within these setups. In the Ventilator Circuit, the inlet is located proximal to the flow sensor, whereas, in the Subject Circuit, the inlet is proximal to the sampling port (as depicted in Figure 2). This discrepancy dictates the rate of CO_2_ level alteration due to the volume within the tubes, as air is propelled forward in fixed tidal increments. The rationale for not placing the gas inlet proximal to the sampling port in the Ventilator Circuit is the need to minimize the distance between the flow sensor and the gas inlet due to the compression of air within the ventilator tube, characteristic of ventilator operation. Otherwise, a substantial disparity arises between the flow sensor's measured flow and the gas delivered by the mass flow controller. Secondarily, the Additional CO_2_ configuration for the selected subject displays considerably less variance in inspired CO_2_ values in comparison to the Reservoir CO_2_ configuration. A closer examination of the data reveals that in the Reservoir CO_2_ configuration, the initial inspired CO_2_ closely approximates the target value but then suddenly declines toward zero. This behavior is expected given our non-excessive usage of fresh gas [8 L/min instead of 15 L/min as proposed by Tancredi et al. (5)]. To avoid negatively biasing the inspired CO_2_ target accuracy of the Reservoir CO_2_ System, we have only tracked the inspired CO_2_ concentrations during the initial phase of inspiration and ignored late declines toward zero. The rationale is that only the initial portion of inspired air is significant, as late inspired air resides in the physiological dead-space. However, since it is not possible from our data to distinguish which portion of the inspired CO_2_ trace belongs to air remaining in the dead-space, there is a potential positive bias of the CO_2_ target accuracy by ignoring the late inspiration phase. Nonetheless, as we are using the Reservoir CO_2_ System as a reference system for the Additional CO_2_ System, any positive bias will not undermine the conclusions drawn regarding the accuracy for the Additional CO_2_ System.

Directing our focus to the aggregated inspired CO_2_ levels (upper portion of Figure 5), it becomes apparent that the Additional CO_2_ method consistently adheres to the target value within the subject dataset. Notably, it performs equally well as the Reservoir CO_2_ method, a comparison further strengthened in Figure 6, which elucidates the mean divergence between measured and target ${\text{F}}_{{\text{CO}}_{2}}^{\text{i}}$. We also see that the end-tidal CO_2_ levels in the lower portion of Figure 5, exhibit small discrepancy between the two methods, motivating our decision to ignore the late inspired CO_2_ level reductions in the Reservoir CO_2_ System. It also highlights that the Reservoir CO_2_ method seems to work well at non-excessive fresh gas flows, as long as the flow is sufficient to cover alveolar ventilation.

In revisiting the upper portion of Figure 5, it is noteworthy that the Additional CO_2_ System consistently undershoots the target value in the Ventilator Circuit. While the offset is relatively small, amounting to < 0.4 percentage points (see Figure 6), understanding the rationale behind this deviation holds intrinsic value. One plausible explanation pertains to the sensitivity of the SFM3200 flow sensor to flow profile. Preliminary assessments suggest that turbulent flow yields higher readings than laminar flow. Consequently, if the ventilator (Primus workstation) produces a higher degree of laminar gas flow relative to the gas used during the calibration of the Additional CO_2_ System (incorporating the flow sensor and mass flow controller), this might elucidate the observed persistent undershoot evident in Figure 5. However, further investigations are requisite to explain this apparent discrepancy in mechanical ventilation. Although any offset is undesirable from a standpoint of repeatability, a consistent target undershoot arguably fares better than a consistent target overshoot concerning subject safety and tolerance.

### 5.2 Resulting oxygen concentrations

Figure 7 shows the inspired and end-tidal O_2_ concentrations for the two subject configurations, using the Additional CO_2_ and Reservoir CO_2_ Systems. As delineated in the Section 2, the Additional CO_2_ method does not actively regulate inspired O_2_ concentration, rather, it manifests as a direct consequence of adding CO_2_ to the inspired gas. Hence, it is unsurprising that the target ${\text{F}}_{{\text{O}}_{2}}^{\text{i}}$ level for the Additional CO_2_ method, depicted by the dashed red line in the upper portion of Figure 7, inversely mirrors the target ${\text{F}}_{{\text{CO}}_{2}}^{\text{i}}$ level (see Figure 3). In the Reservoir CO_2_ method, O_2_ levels are actively controlled by the system and have been maintained at a constant 21 %, as indicated by the dotted red line in Figure 7. To facilitate a comparison between the two methods, the measured and target ${\text{F}}_{{\text{O}}_{2}}^{\text{i}}$ values have been scaled by their baseline value. Given the conspicuous dissimilarities in measured ${\text{F}}_{{\text{O}}_{2}}^{\text{i}}$ levels, one might reasonably anticipate notable discrepancies in end-tidal O_2_ levels. However, a close examination of the lower segment of Figure 7 reveals a lack of pronounced differentiation between the two methods. This phenomenon arises from the recognition that inspired concentration is not the sole determinant of end-tidal values. Variations in alveolar ventilation, by increased or decreased breath frequency and depth, will typically affect end-tidal O_2_ [(and CO_2_), see Equations 1, 2]. Inspecting the end-tidal O_2_ curve for the Reservoir CO_2_ dataset reveals a progressive elevation over time, even though the inspired O_2_ concentration stays fixed, signifying an increasing alveolar ventilation as the experiment unfolds. This seems reasonable given the automatic triggering of reflexes to stimulate deeper and more frequent breaths when CO_2_ is inspired (20). Similarly, in the Additional CO_2_ configuration, end-tidal O_2_ levels appear to rise as the experiment progresses, even though inspired O_2_ transiently decreases. Hence, the variations in alveolar ventilation obscures the disparities between the Additional CO_2_ and Reservoir CO_2_ methods. Advanced control systems, such as prospective end-tidal targeting, account for these changes in alveolar ventilation to provide a more precise and reproducible stimulus (8).

It is worthy of note that during the mechanical ventilation of the test lung, tidal volumes, and consequently alveolar ventilation, remained constant when the test lung was ventilated using pressure-control inflation, but not when volume-control was employed. This discrepancy is understandable since, in volume-control ventilation, the ventilator administers a predefined tidal volume, with any additional CO_2_ gas adding to this volume. Conversely, pressure-control ventilation involves the establishment of a fixed inspiration pressure (P_insp_) at the outset of each breath, maintained for a predetermined duration (T_insp_). In such scenarios, tidal volume becomes dependent solely upon P_insp_, T_insp_ and the compliance of the test lung (or patient), why the addition of CO_2_ gas does not alter the administered volume. Hence, in the practical application of mechanical ventilation in patients using pressure-control, a reduction in end-tidal O_2_ levels is to be anticipated when employing the Additional CO_2_ method to administer CO_2_.

### 5.3 Compatibility with magnetic resonance imaging

We examined BOLD-CVR in two research subjects to evaluate the compatibility of the Additional CO_2_ method with simultaneous MRI measurements. The dataset depicted in Figure 8 presents initial findings, serving as an illustrative demonstration of the feasibility of our proposed Additional CO_2_ method. It is crucial to underscore, nonetheless, that a more extensive, in-depth inquiry is imperative to assess the applicability of the Additional CO_2_ method in an MRI context. For a more detailed exploration of the BOLD-CVR experiment, we direct interested readers to Section 2 of the Supplementary material.

### 5.4 Limitations

Currently, the Additional CO_2_ System only allows targeting of inspired CO_2_. However, in CVR experiments, it is the alveolar CO_2_ concentration which is the parameter of interest. Thus, direct targeting of alveolar CO_2_ would be preferable (4). In the last paragraph of Section 2.1, we have outlined potential enhancements to the Additional CO_2_ method to enable targeting of alveolar O_2_ and CO_2_.

Our Reservoir CO_2_ System had some inherent limitations, particularly the flow of fresh gas which was limited due to technical factors. We used 8 L/min of fresh gas, arguing that this leads to a minimal usage of gas which is comparable to the Additional CO_2_ System. However, as highlighted by Tancredi et al. (5), the Reservoir CO_2_ method performs better with increased gas flow. To partially address this limitation, we adjusted how we computed inspired CO_2_ levels for the Reservoir CO_2_ System, disregarding late CO_2_ level reductions, motivated by the fact that only the initial inspired gas reaches the alveolar space.

In comparing the Additional CO_2_ and Reservoir CO_2_ Systems, we only considered spontaneous breathing. A more comprehensive analysis would also include comparisons in mechanical ventilation. However, this requires more complicated breathing circuits for the reservoir approach, such as those detailed in Winter et al. (9) or Venkatraghavan et al. (10), which were not available to us.

When evaluating the Additional CO_2_ System in mechanical ventilation, we only used a test lung, with no human subject being ventilated. Furthermore, our investigation only considered two types of ventilation mode: volume-control and pressure-control.

## 6 Conclusion

The contemporary landscape of CVR research predominantly features investigations conducted in spontaneously breathing subjects, with limited attention directed toward patients undergoing mechanical ventilation. A notable constraint contributing to this disparity resides in the lack of suitable apparatus for executing CO_2_ gas challenges within a ventilator-dependent setting. Consequently, CVR assessments in ventilated patients have conventionally resorted to alternative stimuli, such as induced apnea, entailing cyclic activation and deactivation of the ventilator, or administration of vasoactive drugs, such as Acetazolamide.

In the present work, we propose a new method, which collaboratively interfaces with mechanical ventilation to administer a variable amount of CO_2_, referred to as Additional CO_2_. We systematically assess the precision of our proposed method in regulating the inspired CO_2_ levels and compare its performance against an established method that relies on a gas reservoir containing a mixture of CO_2_ at varying concentrations. Furthermore, we evaluate the compatibility of our devised system within an MRI environment, conducting a BOLD-CVR study. Our findings support the efficacy of our method in maintaining precise inspired CO_2_ levels in both mechanical ventilation and in spontaneous breathing. Moreover, it can integrate with an MRI scanner to generate BOLD-CVR maps.

While traditional methods using gas reservoirs remain beneficial for populations predominantly composed of spontaneously breathing patients due to their simplicity, our Additional CO_2_ method presents a viable alternative in cases involving both mechanically ventilated and spontaneously breathing patients. This new method avoids the complexities and modifications required by reservoir-based approaches in ventilator circuits. We anticipate that our findings will encourage further CVR research in mechanically ventilated patients in the near future.

## Data availability statement

The raw data supporting the conclusions of this article will be made available by the authors, without undue reservation.

## Ethics statement

The studies involving humans were approved by Swedish Ethical Review Authority (reference number: 2021-04825). The studies were conducted in accordance with the local legislation and institutional requirements. The participants provided their written informed consent to participate in this study.

## Author contributions

GM: Conceptualization, Data curation, Investigation, Methodology, Software, Validation, Visualization, Writing – original draft, Writing – review & editing. ME: Funding acquisition, Project administration, Resources, Supervision, Writing – review & editing. CG: Supervision, Writing – review & editing. GC: Funding acquisition, Supervision, Writing – review & editing. LT: Supervision, Writing – review & editing. AT: Funding acquisition, Project administration, Resources, Supervision, Writing – review & editing.
