# Supplementary material for: High inspired CO2 target accuracy in mechanical ventilation and spontaneous breathing using the Additional CO2 method
Source: Front Med (Lausanne). 2024 May 22;11:1352012. doi: 10.3389/fmed.2024.1352012 (PMC11150593; doi:10.3389/fmed.2024.1352012)
Supplement: Supplementary file 1 [file Data_Sheet_1.pdf]

Supplementary Material

1 SYSTEM DESCRIPTIONS AND COMPONENTS

Figure S1 illustrates the configuration of the Additional CO_2 System (on the left) and the Reservoir CO_2 System (on the right) within a magnetic resonance imaging (MRI) environment. In the Additional CO_2 configuration, certain integral components, namely the flow sensor, mass flow controller, and control unit, are positioned within the confines of the MRI scanner room. In the Reservoir CO_2 configuration, all associated components are situated external to the MRI scanner room.

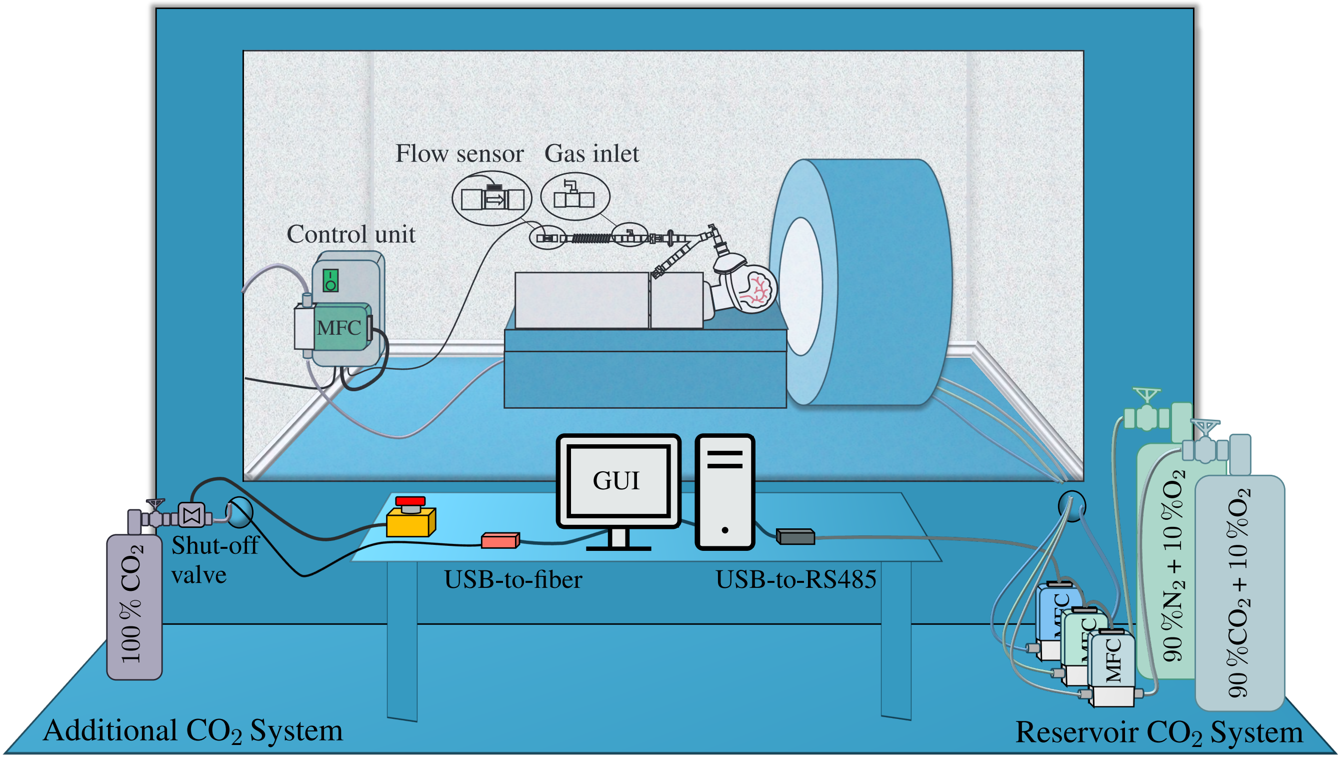

Figure S1. Illustrating the Additional CO_2 and Reservoir CO_2 Systems within an MRI environment. In the Additional CO_2 configuration, the flow sensor, mass flow controller (MFC), and control unit are situated within the confines of the MRI scanner room. This placement choice is required by the necessity to maintain a minimal CO_2 tube length between the MFC and the inlet in the breathing circuit to ensure precise targeting, see main article figure 2. The MFC and control unit are affixed to the wall, while the flow sensor is affixed to the breathing circuit, which is positioned on the scanner board. It is worth noting that the flow sensor remains external to the MRI scanner opening, its position adjusted by the expandable tube in the breathing circuit. A computer-based graphical user interface (GUI) and a $100\,\%$ CO_2 gas cylinder are both positioned outside the scanner room, connected via tubing and optical cables that traverse through wave guides into the scanner room. Furthermore, the figure illustrates the presence of an emergency shut-off valve, which provides the means for rapid termination of the CO_2 supply. In the Reservoir CO_2 configuration, conversely, all equipment is situated external to the scanner room, with only the gas tubes extending into the room through wave guides to establish connections between the MFC:s and the breathing circuit. The expandable tube in this configuration is extended to a length of $2\,\mathrm{m}$, serving as a reservoir. Notably, the flow sensor is not used or attached to the breathing circuit in the Reservoir CO_2 configuration.

1.1 Additional CO_2 System

The Additional CO_2 System is made of various components, which may be categorized into the following main components: gas supply of CO_2, gas control, control unit, and graphical user interface (GUI). These components, along with their respective subcomponents, are detailed in table 1.1 and are visually depicted in the left portion of figure S1. The gas supply is consists of a gas cylinder with $100\,\%$ pure CO_2, affixed to a shut-off valve, thereby facilitating rapid cessation of gas flow to the mass flow controller. The control unit, housing a microcontroller and power supply, controls the setpoint of the mass flow controller via a RS485 databus and reads the flow from the flow sensor through I2C communication to enable a proportional flow of CO_2 gas. The required proportional flow to achieve a desired target CO_2 concentration in the inspired gas is calculated at the GUI and communicated to the control unit via a fiber optic link using a custom data protocol.

1.2 Reservoir CO_2 System

The Reservoir CO_2 System comprises three fundamental components: gas supply of CO_2/N_2/O_2, gas control, and GUI. These constituent elements, along with their respective subcomponents, are detailed in table 1.2 and depicted on the right-hand side of figure S1. The gas supply consists of three gas sources containing $90\,\%$ CO_2 + $10\,\%$ O_2, $90\,\%$ N_2 + $10\,\%$ O_2 and $100\,\%$ O_2, respectively, and are connected to mass flow controller to enable precise formulation of gas mixtures. The setpoint for each mass flow controller is calculated at the GUI, given a total gas flow and target O_2/CO_2 concentrations, and communicated over a RS485 databus. The outlets of the mass flow controllers are attached to tubes which are combined using a 4-way connector before being attached to the gas inlet of breathing circuit, see figure 2 in the main article.

1.3 Breathing circuits

The constituents of the two respiratory circuits employed: Ventilator Circuit and Subject Circuit, are depicted in figure 2 of the main article and listed here in table 1.3.

2 BOLD-CVR EXPERIMENT

The final phase of our investigation entailed a test-retest examination of blood oxygenation level dependent cerebrovascular reactivity (BOLD-CVR) with the aim of evaluating the Additional CO_2 method within an MRI environment and qualitatively compare it to the Reservoir CO_2 method. The experimental configuration is depicted in figure S1.

2.1 MRI protocol

A $3\,\mathrm{T}$ Siemens Prisma Magnetic Resonance scanner was employed to monitor alterations in cerebral blood flow resulting from the inspiration of carbon dioxide by the subjects. The MRI protocol encompassed the acquisition of an anatomical T1-weighted MPRAGE scan for image registration. Parameters for this scan were as follows: flip-angle = $8°$, TR = $2.3\,\mathrm{s}$, TE = $2.36\,\mathrm{ms}$, TI = $0.9\,\mathrm{s}$, GRAPPA = 3, reference lines = 24, voxel-size = $0.9\,\mathrm{mm} \times 0.87\,\mathrm{mm} \times 0.87\,\mathrm{mm}$, and matrix = $208 \times 288 \times 288$. For each CVR run, utilizing either the Additional CO_2 or Reservoir CO_2 technique, a $T2^*$-weighted multi-band (MB) gradient-echo planar imaging sequence (EPI), as provided by the Centre for Magnetic Resonance Research (CMRR, University of Minnesota, USA) was collected (Moeller et al., 2010; Feinberg et al., 2010; Xu et al., 2013). The parameters for this sequence were: flip-angle = $60°$, TR = $1\,\mathrm{s}$, TE = $12.60\,\mathrm{mm}$, $29.88\,\mathrm{mm}$, $47.14\,\mathrm{mm}$ and $64.42\,\mathrm{mm}$, MB-factor = 4, GRAPPA = 2, reference lines = 24, voxel-size = $3\,\mathrm{mm} \times 3\,\mathrm{mm} \times 3\,\mathrm{mm}$, matrix = $68 \times 68 \times 44$, and #measurement = 345. Additionally, single-band reference images (SBRefs)

Table S1. Products and manufacturers of components used in the Additional CO_2 System.

Component		Product, Manufacturer	Comment
Gas supply of CO_2	Gas cylinder	S5 (AL), AirLiquide	$100\,\%$ CO_2, volume $5\,\mathrm{L}$.
	Pressure regulator	Medireg, Linde Gas	One stage regulator, outlet pressure $4.5\,\mathrm{bar}$.
	High pressure tube	CO_2 hospital tube, Linde Gas	Tube between pressure regulator, shut-off valve and mass flow controller.
	Quick-connectors	P/N DQSA/DQBA-M-4N-K6-SA, DK-LOK	Quick connectors with checker valves for easy disconnection of the tube between the shut-off valve and mass flow controller.
Emergency shut-off	3/2 way valve	P/N 126149, Burkert	The 3/2 way valve is connected to outlet of the pressure regulator, and upon breaking the power supply by pressing the stop button, it closes.
	Stop button	P/N XALK178F, Schneider	
Gas control	Mass flow controller	SFC5500, Sensirion	Calibrated and connected to CO_2 gas source. Max. flow: $10\,\mathrm{L\,min^{-1}}$. Communcation via RS485.
	Low pressure tube	Tubclair Al, Tricoflex	Tube between MFC and gas inlet of breathing circuit. ID: $2\,\mathrm{mm}$, OD: $4\,\mathrm{mm}$.
	Flow sensor	SFM3200, Sensirion	Flow range: -100 to $250\,\mathrm{L\,min^{-1}}$. Communication via I2C.
	Quick-connectors	P/N PMC2404/PMCD1004, Colder	Quick connectors with checker valve for easy disconnection of the tube connected to the outlet of the mass flow controller.
Control unit	Microcontroller	Arduino Beetle, DFRobot	Controlling the mass flow controller and flow sensor and communication with GUI.
	Software	Arduino program, Gustav Magnusson (author)	In-house developed.
	UART-optic-converter	SPX17510, Sparkfun	To enable fiber communication between Microcontroller and GUI.
	Power supply	RPT-60, Mean Well	Power supply for components in Control unit as well as the mass flow controller and flow sensor
GUI	Computer	MacBook Pro, Apple	Any personal computer with Python installed can be used as computer.
	Software	Python program, Gustav Magnusson (author)	
	USB-fiber-converter	SPX17508, Sparkfun	The USB converter connects to the computer to enable communication with the Control unit over fiber.
	Fiber optic cable	P/N 164-4255, RS PRO	

were saved. Between each CVR run, the subjects was taken out of the scanner and asked to sit up and rest for a couple of minutes before continuing.

2.2 CO_2 protocol

A simplified CO_2 stimulation protocol was used, comprising a block-stimulus design with an initial baseline period of $1\,\mathrm{min}$, followed by three alternating cycles of $5\,\%$ and $0\,\%$ CO_2 over $45\,\mathrm{s}$ intervals, concluding with an additional minute of baseline recording. To monitor CO_2 and O_2 levels, a Philips Expression MR400 and its IP5 monitor were employed. The end-tidal CO_2 values were made accessible via the RS232 port of IP5 monitor, operating at a data rate of $1\,\mathrm{Hz}$. The recorded end-tidal CO_2 values were subsequently used for the computation of CVR maps based on the BOLD signal acquisitions.

2.3 Flow sensor interference

An unforeseen complication emerged concerning interference of the Additional CO_2 System's flow sensor, positioned within the MRI scanner room. This interference led to disruptions in the communication between the flow sensor and the control unit at the onset of scanning. Further investigation revealed that an alteration in the length of the cable connecting the flow sensor to the control unit had increased its susceptibility to electromagnetic interference from the MRI scanner. Initial pilot trials employed a shorter $2\,\mathrm{m}$ cable without encountering interference issues, but for subsequent scans, a longer $4.7\,\mathrm{m}$ cable was employed to prevent people tripping over the cable. Unfortunately, this extended cable length closely approximated a multiple of the wavelength of the MRI scanner's RF-field ($2.36\,\mathrm{m}$). By shortening the cable to $4.3\,\mathrm{m}$, most of the disturbances were mitigated, although not eliminated. By carefully securing the cable along the scanner bore, a stable communication link between the microcontroller and the flow sensor was achieved. Two subjects were successfully scanned using this setup. However, when attempting to scan a third subject, the interference issues resurfaced, and we ended up settling on only acquiring BOLD-CVR maps in these two subjects.

Following these experiments, the flow sensor cable was again shortened to $2\,\mathrm{m}$, and since then, we have not experienced any further interference issues. While the precise cause of the interference remains unclear, it is evident that the cable length plays a crucial role.

2.4 Preprocessing of MRI data

The MR data was preprocessed using the automated pipeline fMRIPrep 23.1.4 (Esteban et al., 2019). The T1-weighted images were corrected for intensity non-uniformity and skull-stripped. Volume-based spatial normalization to standard space (MNI152NLin2009cAsym, Fonov et al. (2009)) was performed through nonlinear registration with antsRegistration from the ANTs toolbox (Avants et al., 2008). For each of the BOLD runs, a reference volume and its skull-stripped version were generated using the SBRefs. Head-motion parameters with respect to this reference (six parameters corresponding three rotation and three translation parameters) was estimated before any spatiotemporal filtering using mcflirt of the FSL toolbox (Jenkinson et al., 2002). BOLD volumes were then slice-time corrected and resampled onto their original, native space, by applying the transforms to correct for head-motion. A $T2^*$ map was estimated from the preprocessed EPI echoes by voxel-wise fitting the maximal number of echoes with reliable signal in that voxel to a monoexponential signal decay model with nonlinear regression. The calculated $T2^*$ map was then used to optimally combine the preprocessed BOLD images across echoes following the method described by Posse et al. (1999). The BOLD reference was then registered to the anatomical T1 space using mri_coreg (FreeSurfer) followed by flirt (FSL) with the boundary-based registration method. The BOLD

volumes were finally resampled into standard space (MNI152NLin2009cAsym) using the ANTs toolbox and the combined BOLD reference to T1 space and T1 space to standard space transformation.

2.5 Generation of CVR maps

The generation of CVR maps entailed the usage of the preprocessed, brain-masked, standard space BOLD data in conjunction with the end-tidal CO_2 values. First, the BOLD data underwent smoothing through the usage of a Gaussian kernel with a full-width-half-maximum of $6\,\mathrm{mm}$. The end-tidal CO_2 time series was then temporally adjusted to the maximum correlation with the global BOLD signal, thereby establishing a solid initial alignment. Subsequently, the cross-correlation between each voxel and the end-tidal CO_2 time series was computed, and the CO_2 time series was further adjusted on a voxel-by-voxel basis. The voxel-specific temporal adjustments were constrained within a range of $\pm 20\,\mathrm{s}$, and the Pearson's correlation p-value, lower-thresholded at 0.05, was used to filter out voxels with low correlation.

The next step involved regressing the voxel time series against the time-shifted, voxel-specific, end-tidal CO_2 time series. To account for motion, the previously computed rotation and translation head-motion parameters, including their temporal derivative and second power (total of 24 parameters), were included in the regression as confounding time series. The resulting end-tidal CO_2 regression coefficient was used to calculate the percentage change in BOLD signal per unit change in end-tidal CO_2, denoted as $\%\Delta\mathrm{BOLD}/\mathrm{mmHg}$, following the methodology elucidated by Liu et al. (2019).

3 VENTILATOR TEST CASES

Fourteen ventilator test cases, prescribed by the European standard ISO 80601-2-12:2020, were incorporated for the purpose of assessing the efficacy and functionality of the Additional CO_2 method within the context of mechanical ventilation. These test cases, including test lung parameters, can be found in table S4.

REFERENCES

Avants, B. B., Epstein, C. L., Grossman, M., and Gee, J. C. (2008). Symmetric diffeomorphic image registration with cross-correlation: Evaluating automated labeling of elderly and neurodegenerative brain. *Medical Image Analysis* 12, 26–41. doi:10.1016/j.media.2007.06.004

Esteban, O., Markiewicz, C. J., Blair, R. W., Moodie, C. A., Isik, A. I., Erramuzpe, A., et al. (2019). fMRIPrep: A robust preprocessing pipeline for functional MRI. *Nature Methods* 16, 111–116. doi:10.1038/s41592-018-0235-4

Feinberg, D. A., Moeller, S., Smith, S. M., Auerbach, E., Ramanna, S., Glasser, M. F., et al. (2010). Multiplexed Echo Planar Imaging for Sub-Second Whole Brain FMRI and Fast Diffusion Imaging. *PLOS ONE* 5, e15710. doi:10.1371/journal.pone.0015710

Fonov, V., Evans, A., McKinstry, R., Almli, C., and Collins, D. (2009). Unbiased nonlinear average age-appropriate brain templates from birth to adulthood. *NeuroImage* 47, S102. doi:10.1016/S1053-8119(09)70884-5

ISO 80601-2-12:2020 (2020). *Medical Electrical Equipment — Part 2-12: Particular Requirements for Basic Safety and Essential Performance of Critical Care Ventilators*. Standard, European Committee for Standardization

Jenkinson, M., Bannister, P., Brady, M., and Smith, S. (2002). Improved Optimization for the Robust and Accurate Linear Registration and Motion Correction of Brain Images. *NeuroImage* 17, 825–841. doi:10.1006/nimg.2002.1132

Liu, P., De Vis, J. B., and Lu, H. (2019). Cerebrovascular reactivity (CVR) MRI with CO2 challenge: A technical review. *NeuroImage* 187, 104–115. doi:10.1016/j.neuroimage.2018.03.047

Moeller, S., Yacoub, E., Olman, C. A., Auerbach, E., Strupp, J., Harel, N., et al. (2010). Multiband multislice GE-EPI at 7 tesla, with 16-fold acceleration using partial parallel imaging with application to high spatial and temporal whole-brain fMRI. *Magnetic Resonance in Medicine* 63, 1144–1153. doi:10.1002/mrm.22361

Posse, S., Wiese, S., Gembris, D., Mathiak, K., Kessler, C., Grosse-Ruyken, M. L., et al. (1999). Enhancement of BOLD-contrast sensitivity by single-shot multi-echo functional MR imaging. *Magnetic Resonance in Medicine* 42, 87–97. doi:10.1002/(sici)1522-2594(199907)42:1⟨87::aid-mrm13⟩3.0.co;2-o

Xu, J., Moeller, S., Auerbach, E. J., Strupp, J., Smith, S. M., Feinberg, D. A., et al. (2013). Evaluation of slice accelerations using multiband echo planar imaging at 3 Tesla. *NeuroImage* 83, 10.1016/j.neuroimage.2013.07.055. doi:10.1016/j.neuroimage.2013.07.055

Table S2. Products and manufacturers of components used in the Reservoir CO_2 System.

Component		Product, Manufacturer	Comment
CO_2 gas source	Gas cylinder	K-sized, AirLiquide	$90\,\%$ CO_2 + $10\,\%$ O_2, volume $50\,L$.
	Pressure regulator	Multistage, QMT	Two stage regulator, outlet pressure set to $1\,bar$
	High pressure tube	Super Nobelair, QMT	Tube between pressure regulator and mass flow controller.
N_2 gas source	Gas cylinder	K-sized, AirLiquide	$90\,\%$ N_2 + $10\,\%$ O_2, volume $50\,L$.
	Pressure regulator	Multistage, QMT	Two stage regulator, outlet pressure set to $1\,bar$
	High pressure tube	Super Nobelair, QMT	Tube between pressure regulator and mass flow controller.
O_2 gas source	Hospital wall outlet	Unknown	$100\,\%$ O_2
	Pressure regulator	Gas outlet regulator, QMT	One stage regulator, outlet pressure set to $1\,bar$.
	High pressure tube	O_2 hospital tube, Linde Gas	Tube between pressure regulator and mass flow controller.
Gas control	Mass flow controllers	SLA5850, Brooks	All three controllers are calibrated and connected to respective gas source. Max. flow: $10\,L\,min^{-1}$. Communication via RS485.
	Low pressure tube	Tygon E3603, Saint-Gobain	Tube between mass flow controllers and 4-way connecter. ID $6.4\,mm$, OD $9.5\,mm$.
	4-way connector	P/N X0-4NK, Colder	Connecting the three mass flow controller tubes to the gas inlet of the breathing circuit
GUI	Computer	MacBook Pro, Apple	Any personal computer with Python installed can be used as computer.
	Software	Python program, Gustav Magnusson (author)	
	USB-RS485-converter	P/N USB-RS485-WE-5000-BT, FTDI Chip	USB converter which enables RS485 communication between computer and mass flow controllers.

Table S3. Products and manufacturers of components used in the breathing circuits for mechanical ventilation, Ventilator Circuit, and spontaneous breathing, Subject Circuit.

Component		Product, Manufacturer	Comment
Ventilator Circuit	Gas inlet	P/N 1947000 + 2710000, Intersurgical	A luer-connector for the mass flow controller in Additional CO_2 System to connect to.
	Humidifier	MR290, Fisher & Paykel	An empty humidifier with no water, to mix gas.
	Connector humidifier	P/N 1992000, Intersurgical	Connecting gas inlet connector to humidifier.
	Coaxial ventilator tube	P/N Q712218N-IGS, Envisen	Standard ventilator tube, $2\,\mathrm{m}$ long.
	Elbow with sampling port	P/N 2714000, Intersurgical	To sample O_2/CO_2 gas.
	Test lung	AccuLung, Fluke Biomedical	Test lung with variable compliance $(10, 20$ and $50\mathrm{mL\,mbar}^{-1})$ and resistance $(5, 20$ and $50\mathrm{mbar\,L}^{-1}\,\mathrm{s})$
Subject Circuit	Expandable tube	P/N 1526000, Intersurgical	Expanded in the Reservoir CO_2 configuration, contracted in Additional CO_2 configuration. ID $22\,\mathrm{mm}$.
	Gas inlet	P/N 1947000 + 2710000, Intersurgical	A luer-connector for the mass flow controllers in Additional CO_2 or Reservoir CO_2 Systems to connect to.
	One-way valve	P/N 1921000, Intersurgical	Two used to control air flow.
	Filter	P/N M1003346, Timik Medical	Particle filter.
	Y-piece	P/N 1901000, Intersurgical	Separates inspiration/expiration side of circuit.
	Outlet adapter	P/N 1967000, Intersurgical	$22\,\mathrm{mm}$-female to $22\,\mathrm{mm}$ female adapter, to connect outlet of Y-piece to the one-way valve.
	Elbow with sampling port	P/N 2714000, Intersurgical	To sample O_2/CO_2 gas.
	Face mask adapter	3D-printed, Gustav Magnusson (author)	$22\,\mathrm{mm}$-female to $30\,\mathrm{mm}$ male adapter.
	Face mask	Mask 7450 V2, Vyaire	Variable sized (XS to L).

Table S4. The 14 ventilator test cases used to evaluate the Additional CO_2 method in mechanical ventilation. Note that the *Test case - Number* refers to tables 201.104 and 201.105 of the European standard ISO 80601-2-12:2020.

Test case		Test lung parameters		Ventilator settings					
Number	Control mode	Compliance $(\mathrm{mL\,mbar^{-1}})$	Resistance $(\mathrm{mbar\,L^{-1}\,s})$	Frequency (breaths/min)	Insp. time (s)	FiO_2 (%)	PEEP (cmH2O)	Insp. pressure $(\mathrm{cmH_2O})$	Tidal volume (L)
1	Volume	50	5	20	1	30	5	–	500
2	Volume	50	20	12	1	90	10	–	500
3	Volume	20	5	20	1	90	5	–	500
4	Volume	20	20	20	1	30	10	–	500
5	Volume	20	20	20	1	30	5	–	300
6	Volume	20	50	12	1	90	10	–	300
7	Volume	10	50	20	1	30	10	–	300
1	Pressure	50	5	20	1	30	5	10	–
2	Pressure	50	20	12	1	90	10	15	–
3	Pressure	20	5	20	1	90	5	25	–
4	Pressure	20	20	20	1	30	10	25	–
5	Pressure	20	20	20	1	30	5	15	–
6	Pressure	20	50	12	1	90	10	10	–
7	Pressure	10	50	20	1	90	5	15	–
